# Commensal *Pseudomonas* protect *Arabidopsis thaliana* from a coexisting pathogen via multiple lineage-dependent mechanisms

**DOI:** 10.1038/s41396-021-01168-6

**Published:** 2021-12-11

**Authors:** Or Shalev, Haim Ashkenazy, Manuela Neumann, Detlef Weigel

**Affiliations:** Department of Molecular Biology, Max Planck Institute for Developmental Biology, 72076 Tübingen, Germany

**Keywords:** Microbial ecology, Plant ecology

## Abstract

Plants are protected from pathogens not only by their own immunity but often also by colonizing commensal microbes. In *Arabidopsis thaliana*, a group of cryptically pathogenic *Pseudomonas* strains often dominates local populations. This group coexists in nature with commensal *Pseudomonas* strains that can blunt the deleterious effects of the pathogens in the laboratory. We have investigated the interaction between one of the *Pseudomonas* pathogens and 99 naturally co-occurring commensals, finding plant protection to be common among non-pathogenic *Pseudomonas*. While protective ability is enriched in one specific lineage, there is also a substantial variation for this trait among isolates of this lineage. These functional differences do not align with core-genome phylogenies, suggesting repeated gene inactivation or loss as causal. Using genome-wide association, we discovered that different bacterial genes are linked to plant protection in each lineage. We validated a protective role of several lineage-specific genes by gene inactivation, highlighting iron acquisition and biofilm formation as prominent mechanisms of plant protection in this *Pseudomonas* lineage. Collectively, our work illustrates the importance of functional redundancy in plant protective traits across an important group of commensal bacteria.

## Introduction

The health of a plant depends to a large extent on its resident microbiota. The effect of individual microbes on plant health has been extensively investigated since the dawn of phytopathology [1], mainly focusing on pathogens, considering their profound effect on global agriculture and food supply [2]. The ability of phytopathogens to overpopulate plants, leading to disease, is reflected in the reciprocal ability of the plant to recognize and control these pathogens [3]. Nonetheless, in recent years there has been an increasing realization that the plant relies not only on its immune system but also on other resident microbes to suppress pathogens [4–7].

For example, in natural settings, the health of the ephemeral plant *Arabidopsis thaliana* is associated with many bacteria that reduce disease threats due to filamentous microbes [5]. Similar patterns have also been found in controlled settings, with suppression of bacterial pathogens by other bacteria [8, 9]. These protective agents can have several modes of action, including (i) activation of systemic defences that spread throughout the plant [10], (ii) outcompeting pathogens over nutrients [11, 12], and (iii) direct antibiosis [13, 14]. These mechanisms are non-exclusive and can operate simultaneously.

These studies have greatly advanced our understanding of protective interactions in plants, even if the investigated pathogens and protective microbes do not always coexist in the wild. A notable exception is a recent study in which individual members of native tomato microbiomes were tested for suppression of the soil-borne pathogen *Ralstonia solanacearum* [15]. This report focused on siderophore production and competition for iron as a known mechanism for microbe-microbe competition, revealing a link to pathogen inhibition in the plant rhizosphere. The work also highlighted how insights can be gained from investigating pathogen protection in a phylogenetic framework [15].

Substantial diversity among *Pseudomonas*, a bacterial genus that includes pathogens, commensals and mutualists of plants (Haas and Défago 2005; Baltrus et al. 2017), was found in a survey in southwest Germany, based on genome sequencing of 1524 isolates isolated from wild *A. thaliana* plants [16]. One cryptically pathogenic lineage dominated this collection, although other, apparently commensal strains were found as well. By employing synthetic communities of commensal and pathogenic *Pseudomonas* from this collection, commensal strains were found to protect *A. thaliana* from pathogenic *Pseudomonas* strains that co-occur in the wild [17]. In a parallel study with a *Pseudomonas* collection from an *A. thaliana* relative, commensal strains were shown to often outcompete pathogenic *Pseudomonas syringae* isolates sampled from the same plant [18].

These studies point to the importance of interactions among wild *Pseudomonas* strains in maintaining plant health. Here, we leveraged a local *Pseudomonas* collection from *A. thaliana* [16] to examine the extent of protection conferred by commensal *Pseudomonas* against co-existing pathogenic *Pseudomonas*, and to discover some of the underlying mechanisms. Using a high throughput image-based assay, we monitored outcomes of systematic co-infections of a diverse set of commensal *Pseudomonas* with a focal *Pseudomonas* pathogen, covering the entire phylogeny of commensal *Pseudomonas* isolates in this collection. We found that protection by commensals was a common feature, although it was enriched in a specific lineage. We discovered bacterial genes for plant protection using genome-wide association and comparative genomics. Using knockout mutants, we experimentally validated the role of several candidate genes in plant protection, establishing a link to iron uptake and biofilm formation in the mitigation of phyllosphere pathogens.

## Results

### Systemic co-infections of commensal *Pseudomona*s with an individual pathogen

To examine the ability of commensal *Pseudomonas* strains to protect host plants from members of a pathogenic *Pseudomonas* lineage, we made use of a local isolate collection [16]. We henceforth refer to an operational taxonomic unit (OTU) as reported in that study as “ATUE” (isolates from Around TUEbingen), and following previous findings [16, 17], we refer to the lineage ATUE5 as pathogenic, and to all non-ATUE5 lineages as commensals.

**Fig. 1:**
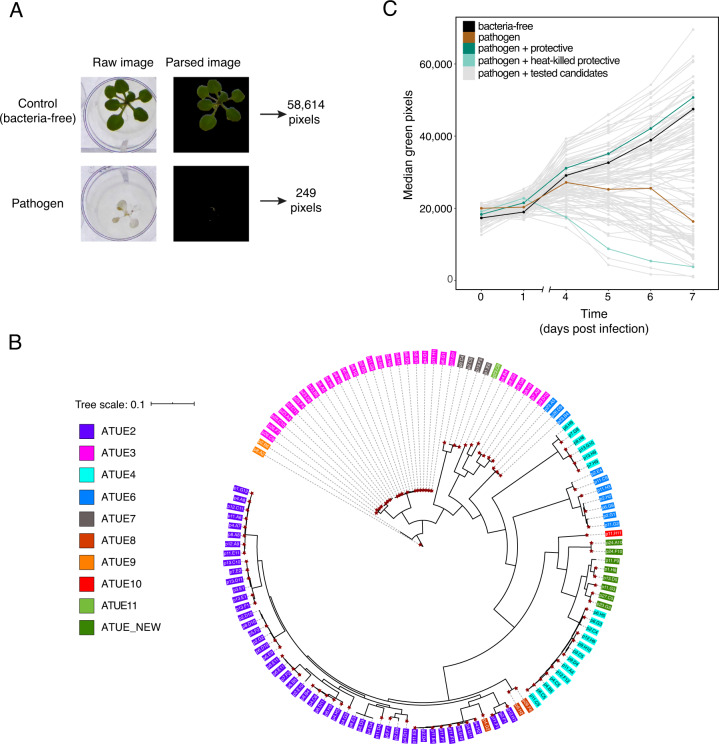
Panel of potentially protective *Pseudomonas* isolates and experimental design. **A** Illustration of image processing to enumerate plant green pixels, approximating plant biomass. **B** Phylogenetic tree of 127 representatives, putatively commensal non-ATUE5 strains sampled from southwest Germany [16]. All other non-ATUE5 isolates in this collection are represented in this core collection by a strain with which they share ≥ 99% of genes. Colors indicate the ATUE group [16], and asterisks the 99 strains used here. **C** Daily median of plant green pixels among the different treatments. Control treatments: Bacteria-free buffer, pathogen only, and co-infections of the pathogen with the protective strain and the heat-killed protective strain. Plants were imaged daily. *n* = 8 replicates per treatment.

We grew plants on MS agar and monitored plant growth and health by extracting the number of green pixels from images over time (illustration in Fig. 1A). Green pixel count and rosette fresh weight were strongly correlated (Supplementary Fig. S1; *R*^2^ = 0.92, *p* value < 2.2e-16), validating the use of green pixels as a proxy for the plant biomass.

We use a conservative definition of plant protection, with protective strains leading to near-normal plant growth (comparable with uninfected plants) in the presence of a pathogen. To estimate how common the ability of non-ATUE5 strains to protect against the impact of pathogenic *Pseudomonas* is, we infected plants with a panel of non-ATUE5 isolates in the presence of a common ATUE5 representative, strain p4.C9, which hereafter will be referred to as “the pathogen” or “the ATUE5 pathogen”. This isolate was chosen because it is dominant over other ATUE5 strains but at the same time highly susceptible to the presence of non-ATUE5 strains, correlating with plant protection [17]. We excluded highly similar isolates, with Jaccard distances ≥ 0.99 in gene content. From a total of 151 non-ATUE5 strains in our local collection [16], we initially chose a subset of 127 isolates, from which we were able to revive 99 (Fig. 1B; Supplementary Table S1). We included three isolates from the pathogenic ATUE5 clade as control (Supplementary Table S1), resulting in 102 strains that were tested in co-infections with the pathogen p4.C9.

One of the 102 strains, p5.F2, was known to suppress ATUE5 strains inside plants [17] and was used as a “protective control”. To confirm that protection was due to bacterial activity and not merely a host response to the inoculum, e.g., PAMP-triggered immunity, we carried out infections with a heat-killed preparation of our protective control. As expected, co-infection with the protective strain resulted in normal plant growth, while treatment with the pathogen by itself or co-inoculation with the heat-killed strain impaired growth (Fig. 1C; Supplementary Fig. S2; mean growth [Δ7dpi-0dpi green pixels]: control 29,227 [15,721, 40,662], pathogen 3328 [−10,350, 14,759], pathogen + heat-killed protective −5222 [−16,410, 6211] and protective 29,585 [16,323, 40,812], at 95% confidence interval).

### Plant protection is phylogenetically widespread, but also lineage-specific

**Fig. 2:**
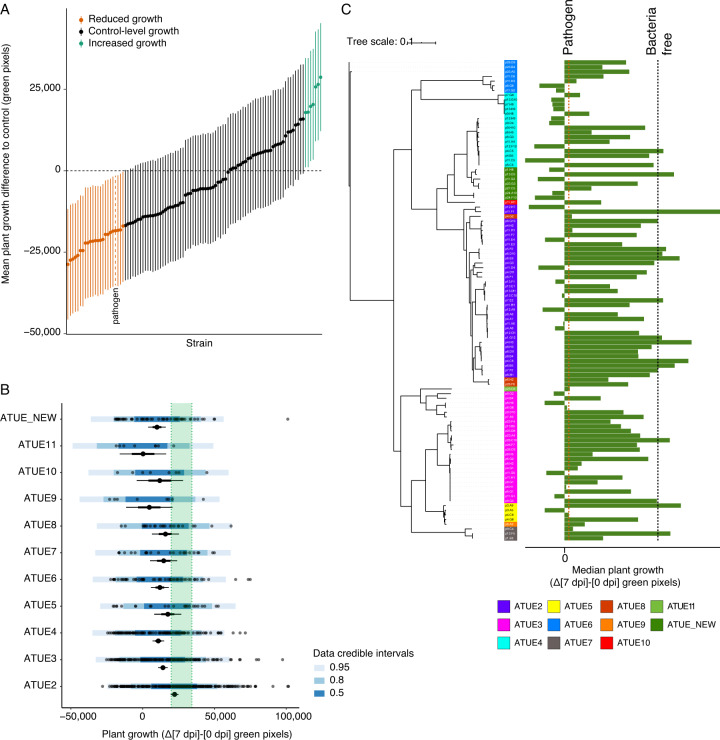
Protection by different *Pseudomonas* strains is common and enriched in the ATUE2 lineage. **A** Mean difference in plant growth to control, after co-infection with different *Pseudomonas* strains and the focal p4.C9 pathogen. Growth was measured as the change in green pixels between days 0 and 7 after infection. Vertical lines indicate 95% Bayesian credible intervals and dots indicate the median estimate. The dashed vertical line signifies the positive control, that is, the pathogen-only treatment and the dashed horizontal line signifies the negative control, that is, the baseline average growth in the absence of bacteria. *n* = 8 replicates each. **B** Plant growth after co-infections, binned by ATUE group. In each ATUE group, raw data for individual replicates are shown with dots; the overlain shades of blue indicate the posterior predictive intervals, as presented on the bottom right. The mean growth for each ATUE group is also shown; the dot indicates the median, the thin horizontal line the 95% credible interval, and the thick line the 67% credible interval. Green shade indicates the 95% credible interval of the negative control, growth in the absence of bacteria. See Supplementary Table S1 for the number of strains in each ATUE group. **C** Median plant growth after co-infections, ordered by phylogeny. Colors indicate ATUE groups [16]. Medians of plant growth in mono-association with pathogen p4.C9 or without infection indicated by dashed vertical lines.

Co-inoculation of non-ATUE5 strains with the ATUE5 pathogen led to a range of outcomes for plant health, from reduced to enhanced growth in comparison to uninfected plants, with protection being common (Fig. 2A).

Protection was unevenly distributed among the three highly sampled ATUE groups—ATUE2, ATUE3, and ATUE4—with ATUE2 being the most protective mean growth (Δ[7 dpi]-[0 dpi] green pixels, 95% CI): ATUE2 22,231 [19,522, 24,908], ATUE3 14,120 [10,780, 17,389], ATUE4 10,868 [6844, 14,937], and bacteria-free 27,069 [19,882, 34,264]) (Fig. 2B).

Within each ATUE group, there was considerable variation in protective ability, even among strains with highly similar genomes (Fig. 2C). In some cases, closely related strains had contrasting activities, with one providing robust protection and the other having no effect, e.g., ATUE2 strains p11.F1 and p12.H7: mean growth after coinfection with p11.F1 being 25,554 pixels [8303, 42,567], but with p12.H7 being −28,914 pixels [−45,789, −11,837]).

Even within ATUE5, some isolates had the protective ability. We had chosen the three ATUE5 strains because in a previous set of experiments [17], they had appeared to be less competitive than our focal ATUE5 pathogen p4.C9, with lower abundance in the context of *A. thaliana* infections with synthetic communities of ATUE5 and non-ATUE5 strains. Surprisingly, two of them also mitigated the pathogen effect, causing normal plant growth. This further exemplifies the differential functions within ATUE groups (Fig. 2C).

### ATUE2 protective genes are lineage-specific

That functional variation could not be explained by phylogeny, considering both topology or branch length, suggested that variation in gene content, possibly due to horizontal gene transfer, was causal for protection. We, therefore, were curious whether the presence/absence (P/A) of gene orthology groups could explain the propensity for plant protection.

**Fig. 3:**
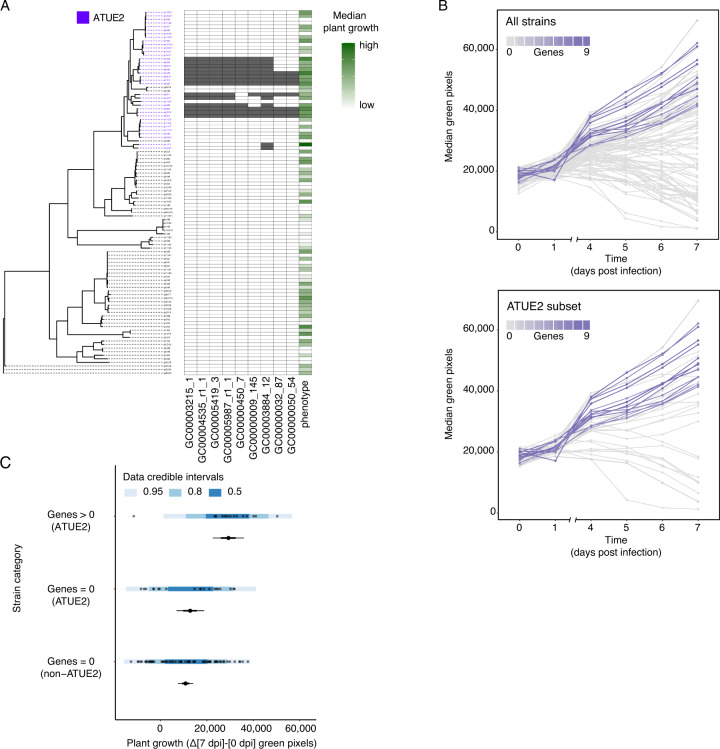
Genes most strongly associated with protection are unique to ATUE2. **A** Presence/absence variation of nine genes that are most strongly associated with protection. Strains are ordered by their phylogeny. Strains belonging to the ATUE2 group are indicated in magenta. Gene presence is indicated in grey, absence in white. Growth was measured as the change in green pixels between days 0 and 7 after infection, with the scale indicated on the right. **B** Daily median of plant green pixels among all strains (top panel) or the ATUE2 subset (bottom panel). Shades of magenta indicate the number of protective genes out of 9 present in each strain, as indicated by the scale on the bottom. Eight replicates per treatment. **C** Plant growth after co-infections with the pathogen p4.C9, binned by (i) the presence of at least one gene from the set of the nine protective genes (presented in panel **A**) in a given commensal strain, and (ii) membership in the ATUE2 group. In each group, raw data for individual replicates are shown as dots; the overlain shades of blue indicate the posterior predictive intervals, as indicated on the bottom right. The mean growth for each group is also shown; the dot indicates the median, the thin horizontal line the 95% credible interval, and the thick line the 67% credible interval. *n* = 8 replicates per strain, with the number of strains in the different categories as follows: non-ATUE2 with 0/9 genes = 67; ATUE2 with 0/9 genes = 20, and ATUE2 with at least 1/9 genes = 16.

There were 32,753 gene orthology groups, that were found in more than one strain, but not shared by all ATUE strains. We examined the correlation between plant growth and the presence of each of the individual orthology groups using treeWAS [19], a tool for genome-wide association studies (GWAS) in bacteria. Because treeWAS accounts for population structure, it will remove true positives that are highly correlated with population structure [20]. To address this issue, we used Spearman’s rank correlation coefficient (SRCC) to search for genes that were correlated with protection. SRCC should also be useful for differentiating a global from a lineage-specific signal. To this end, we calculated SRCCs separately among the highly sampled ATUE2, ATUE3, and ATUE4 groups, considering the difference in median green pixels between 0 and 7 dpi. For treeWAS, we used three additional plant growth metrics: the median change in green pixels between the last day of the experiment (7 days post-infection) to the day of infection (‘‘median growth’’), a Bayesian-derived approximation of the ‘median growth’’ (‘‘cdl50’’, i.e., the posterior distribution median of the ‘median growth’’), a binary categorization based on cdl50 and the area under the curve while accounting for all sampled time points (Supplementary Table S2; Methods). The extent of agreement between the four phenotypes was used as another indicator for the robustness of each association (Supplementary Table S2). We overlapped the results from all four analyses and removed genes with a negative SRCC (SRCC < 0, which implies negative effects on plant health in addition to those of the pathogen rather than protection from the pathogen), leaving us with 14 strong candidates for plant protection (Supplementary Table S2). The nine genes with the highest SRCC values (Rho 0.37-0.46) were unique to ATUE2 (Fig. 3A).

The existence of a gene set unique to one lineage implies that plant protection by *Pseudomonas* is not driven by a universal mechanism in this genus, but rather by lineage-specific processes. To test this assumption, we ran the same GWAS analysis on each of the main ATUE groups separately, and independently analyzed the subsets of strains related to ATUE2, ATUE3, and ATUE4. In total, 95 genes with positive SRCCs were significantly associated with plant protection: 14 genes from the full set of strains, 46 from ATUE4, 35 from ATUE3, and none from ATUE2. Except for a single gene, we found no overlap among hits from the different ATUE groups, providing further evidence for lineage specificity (Supplementary Fig. S3).

Half of all protection-associated genes, 47 out of 95, were annotated as encoding ‘uncharacterized protein’’ or had no-hit in TrEMBL and Swiss-Prot databases (Supplementary Table S2). Out of the well-annotated genes, a few seemed to be likely to be directly related to microbe-microbe interactions: three iron-uptake-related genes unique to ATUE2 (GC00000450_7: ‘TonB_C domain-containing protein’’, GC00000032_87: ‘Putative iron(III) dicitrate sensor protein FecR’’ and GC00000050_54: ‘Probable RNA polymerase sigma factor FecI’’), a gene related to resistance to antimicrobial peptides in ATUE3 (GC00000089_55: ‘UDP-4-amino-4-deoxy-L-arabinose–oxoglutarate aminotransferase’’ [*arnB*] [21]), and an antitoxin gene in ATUE4 (GC00007392_r1_1: ‘Antitoxin FitA’’) alongside mobility-related genes (GC00002204_5: ‘Twitching motility protein PilT’’, GC00000715_r1_r1_2: ‘Pilus assembly protein PilW’’). These putative functions suggest not only a diverse set of genes but also diverse protective mechanisms among commensal *Pseudomonas*.

Several of these genes had identical SRCC values in a specific ATUE group and turned out to be physically linked (Supplementary Table S2; details in Methods). There was also evidence for genetic elements associated with horizontal gene transfer, such as phage genes (clusters 3 and 4 in Supplementary Table S2), consistent with these clusters being genomic islands. Cumulatively, these results suggest a scenario in which plant protection by commensal *Pseudomonas* is driven by multiple, clade-specific mechanisms that were horizontally transferred.

As described above, of the 14 protection-associated genes found among all strains, nine were unique to ATUE2 (Fig. 3A). Although there were no significant hits when we analyzed the ATUE2 subset on its own, strains carrying these nine genes were more protective not only when compared among all strains, but also within ATUE2 (Fig. 3B, C). For ATUE2 strains with at least one of these genes, plant growth was 29,299 pixels (22,567, 35,983, 95% confidence interval). In contrast, plant mean growth for ATUE2 strains without any of these genes was only 12,780 pixels (6891, 18,821), and for non-ATUE2 strains without any of these genes, it was only 10,791 pixels (7541, 14,078). Moreover, we found that the effect of these nine genes was additive within ATUE2, and plant growth was correlated with the number of genes present in a given *Pseudomonas* strain (Supplementary Fig. S4; *R*^2^ = 0.55, *p* value = 0.0004). These results highlight the importance of these nine genes in plant protection both among all *Pseudomonas* strains in our collection, and within ATUE2, despite the lack of statistical signal in the ATUE2-subset alone.

### Plant protection by ATUE2 linked to iron acquisition and biofilm formation

We selected the nine ATUE2-lineage specific candidates for gene deletion in the protective strain p5.F2 as a representative for the ATUE2 clade, to validate the role of these genes in plant protection. Since two genes were found next to each other (GC00000032_87 and GC00000050_54), we included them in one deletion, resulting in eight mutations. Because our knockout mutants were marked with gentamicin resistance, we generated a wild type strain that also expresses gentamicin (as well as the lux operon), ensuring a similar metabolic burden (hereafter “wild type” or “WT”; see Methods).

**Fig. 4:**
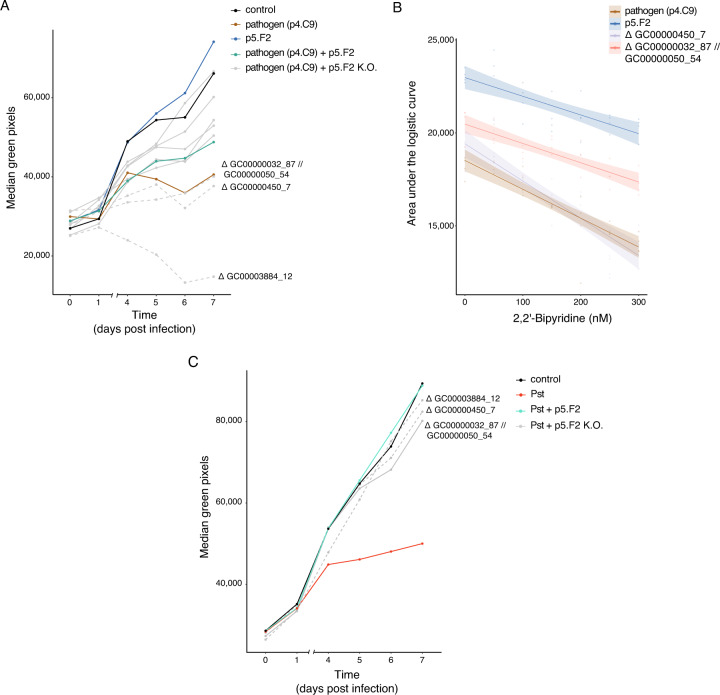
Functional analysis of genes implicated in plant protection. **A** Daily median of plant green pixels after treatment with control, p4.C9 pathogen, the protective strain p5.F2, a mixture of the pathogen and p5.F2, and mixtures of the pathogen and each of eight p5.F2 knockout mutants, indicated as p5.F2 K.O. Dashed lines indicate three treatments with p5.F2 knockout mutants that lost their ability to protect the plant (Δ GC00000032_87 // GC00000050_54, Δ GC00000450_7, and Δ GC00003884_12), as analyzed in Supplementary Fig. S5A. *n* = 20 replicates. A second experiment gave similar results, as detailed in Supplementary Fig. S6A, B. **B** In vitro growth of the pathogen, p5.F2 and two knockout mutants (Δ GC00000032_87 // GC00000050_54 and Δ GC00000450_7) as a function of 2,2’-bipyridine concentration, analyzed in six 50 nM increments from 0 to 300 nM. OD_600_ was monitored for 10 h (see also Supplementary Fig. S5 and **Methods**). The logistic area under the growth curve was extracted as a proxy for bacterial growth. The shaded area indicates 95% confidence intervals of the regression curve. *n* = 4 for each bacterial strain grown in the corresponding 2,2’-bipyridine concentration. **C** Daily median of plant green pixels after treatment with control, *Pseudomonas syringae* pv. *tomato* DC3000 (*Pst*), mixture of *Pst* and p5.F2, and mixture of *Pst* and each of the three tested p5.F2 knockout mutants, indicated as p5.F2 K.O. Dashed lines indicate two treatments with p5.F2 knockout mutants that lost their ability to protect the plant (Δ GC00000032_87 // GC00000050_54 and Δ GC00000450_7), as analyzed in Supplementary Fig. S5B. *n* = 20 replicates. A second experiment gave similar results, as detailed in Supplementary Fig. S6C, D.

Out of the eight knockout mutants, three lost their ability to mitigate the effect of the pathogen p4.C9 following co-infection, and we call them henceforth “non-protective mutants” (Fig. 4A; Supplementary Fig. S5A; results of an additional experiment with the three non-protective mutants in Supplementary Fig. S6A, B). These three non-protective mutants did not affect plant weight when tested individually, similarly to the wild type (Supplementary Fig. S7A, B), implying that the weight reduction after co-infection was due to the pathogen or to the interaction with the pathogen, rather than the knockout mutants themselves.

Out of the four genes in the three mutants that had lost their ability to protect from pathogens, three adjacent genes are annotated as iron-related: one encodes a TonB_C domain-containing protein (GC00000450_7) [22, 23], one a putative iron (III) dicitrate sensor related to FecR (GC00000032_87) [24], and one a probable RNA polymerase sigma factor related to FecI (GC00000050_54) [25] (SupplementaryTable S2).

The fourth gene, GC00003884_12, is annotated as encoding an ‘uncharacterized protein’’ (Supplementary Table S2). When ΔGC00003884_12 was grown in tubes while shaking, clumps formed, suggesting the aberrant formation of cellular aggregates. To investigate this further, we grew the wild type and ΔGC00003884_12 overnight, and then let the cell suspension settle on the benchtop. After 1 h, cell sediment had formed in the ΔGC00003884_12 tube, with a clear liquid forming at the top and aggregates at the bottom (Supplementary Fig. S8), while the wild-type cells had stayed in suspension and the culture still had retained its homogenous opaque appearance. This phenotype implies that at least in the tested conditions, GC00003884_12 suppresses cell-cell aggregation and biofilm formation.

To validate the role of GC00000032_87 // GC00000050_54 and GC00000450_7 in iron uptake, we performed an in vitro iron-deficiency growth assay. The wild-type strain, the ΔGC00000032_87 // GC00000050_54 and ΔGC00000450_7 mutants and the pathogen were grown in LB with increasing levels of the iron chelator 2,2’-dipyridyl. We also tested ΔGC00003884_12 in the same assay, but noted an unusual growth curve shape for this strain, regardless of chelator levels (Supplementary Fig. S9), in agreement with the formation of cell aggregates we had observed (Supplementary Fig. S8). Consequently, we excluded the mutant ΔGC00003884_12 from further analysis.

Both ΔGC00000032_87 // GC00000050_54 and ΔGC00000450_7, as well as the pathogen p4.C9, grew more slowly than the wild-type commensal strain in LB without chelator (Fig. 4B; Supplementary Figs. S9, S10A; mean difference to control: pathogen −4424 AUC [−5299, −3551, 95% confidence interval], ΔGC00000450_7 −3526 AUC [−4412, −2634] and ΔGC00000032_87 // GC00000050_54 -2,469 AUC [−3337, −1587]). This confirms that both loci have a role in growth that is independent of iron availability. Increasing chelator levels led to a reduction in the growth of the mutants, the pathogen and the wild-type commensals, with the pathogen and ΔGC00000450_7 being more sensitive to the iron deficiency than the other three strains (Fig. 4B; Supplementary Figs. S9, S10B; mean slope difference to control: pathogen −5.6 AUC [−10.5, −0.7, 95% confidence interval], ΔGC00000450_7 −10.1 AUC [−15.1, −5.3] and ΔGC00000032_87 // GC00000050_54 -0.4 AUC [−5.3, 4.3]). These findings provide evidence for the involvement of iron acquisition in plant protection by ATUE2 strains and specifically imply that ATUE2 strains protect the plant by outcompeting the pathogen over iron.

Taken together, these results suggest that ATUE2 members can protect *A. thaliana* from coexisting pathogenic ATUE5 isolates via mechanisms related to iron uptake, and apparently also to biofilm formation. To test whether these mechanisms act specifically against the local ATUE5 strains or whether they are also effective against other *Pseudomonas* pathogens, we co-infected the protective wild-type commensal and the three non-protective mutants with the model pathogen *Pseudomonas syringae* pv. *tomato* DC3000 (*Pst*) [26]. *Pst*-like strains were rare in the local *A. thaliana* populations from which ATUE2 was sampled [16], hence it is unknown whether ATUE2-like strains and *Pst*-like strains co-exist. The wild-type commensal protected against *Pst* infection, greatly reducing the effects of *Pst* on plant growth (Fig. 4C; Supplementary Fig. S5B). While this suggests that ATUE2 protective ability is not restricted to the pathogenic lineage ATUE5, the exact modes of protection against the two pathogens seem to differ, since our mutants that had lost protection against the ATUE5 pathogen were still able to protect against *Pst* infection (Fig. 4C; Supplementary Fig. S5B; replicated in an additional experiment, shown in Supplementary Fig. S6C, D).

## Discussion

Here, we systematically tested the ability of non-pathogenic *Pseudomona*s strains to protect their natural host from a pathogenic *Pseudomona*s lineage, using a set of commensal and pathogenic isolates that co-exist in nature. We found that plant protection is a common function among non-pathogenic *Pseudomonas* lineages, but with significant differences even between closely related strains. A genome-wide association study (GWAS) pointed to lineage-specific gene sets associated with plant protection. Focusing on a small set of candidate genes, we demonstrated that iron acquisition and biofilm formation appear to play an important role in helping protective *Pseudomonas* strains to mitigate the impact of pathogenic *Pseudomonas* in the phyllosphere.

While protective and non-protective strains are found in several ATUE clades, the fraction of either type varies between lineages. Similar patterns were described in a recent study focused on bacterial commensals that protect from the effects of *Ralstonia solanacearum*, a tomato pathogen [15]. In addition, a few strains enhanced disease outcomes in co-infection trials. One plausible explanation is that some strains that are closely related to commensals facilitate the activity of the pathogen, as described for fungi that modify the response of *Populus trichocarpa* to the leaf rust pathogen *Melampsora* [27]. Alternatively, some ATUE strains might actually be pathogenic by themselves despite clustering phylogenetically with true commensals. This would not be without precedence, since a pathogenic lineage within a commensal clade has been described for plant-associated *Pseudomonas* [28]. The two scenarios—pathogenesis-facilitating commensals and pathogenic strains within a commensal clade—do not have to be mutually exclusive, and they clearly warrant further investigation to reveal the underlying genes, physiological mechanisms and evolutionary forces.

The involvement of iron uptake suggests competition between commensal and pathogenic *Pseudomonas* over this micronutrient as a potential mechanism for plant protection. This is in agreement with the importance of iron competition in microbial communities [15, 29–32] and the link between iron availability and plant pathogenesis [33, 34]. Iron, an essential micronutrient [35], has limited bioavailability [36], driving competition for iron and evolutionary diversification of iron uptake instruments [37]. Therefore, it is not unexpected that iron competition dictates microbial interactions within the plant, which in turn can affect host health. In particular, TonB receptors, which are involved in siderophore-mediated iron uptake [38], are important vehicles for nutrient uptake in phyllosphere bacteria [39]. This further supports our finding of an iron-uptake TonB receptor gene as responsible for plant protection.

A new link that we discovered is a connection between bacterial biofilm formation and plant protection. Bacterial aggregation affects many bacterial activities, including altered competition with other bacteria [40]. Therefore, it is perhaps not surprising that biofilm formation can influence the competition between commensal and pathogenic *Pseudomonas* in the plant. More insight into the role of biofilms in pathogen protection will come from microscopic imaging of bacterial growth and bacteria-bacteria interactions during infections.

We have used comparative genomics to pinpoint protective genes among various *Pseudomonas* commensal lineages. Our results imply that the underlying protective mechanisms are lineage-specific, based on the annotation of GWAS hits. An additional complexity is that different genetic and physiological mechanisms appear to be responsible for protection from different pathogenic *Pseudomonas* lineage. This observation in turn warrants further investigation of the exact processes by which the natural pathogen ATUE5 pathogen and the model pathogen *Pst* reduce growth and/or cause disease in *A. thaliana*. Our observations underscore the benefits of studying diverse natural pathogens, commensals and mutualists of wild plants. From an applied perspective, more knowledge of the complete set of tools by which plants are being attacked by bacteria and other microbes may also lead to new avenues for how to protect crop plants from their enemies.

With the candidate protective genes that we discovered from GWAS, one can now return to the natural *A. thaliana* populations from with the commensal and pathogenic *Pseudomonas* strains were sampled [16], and investigate the prevalence of these genes, their co-occurrence, and also their impact on levels of colonization by pathogenic *Pseudomonas* in the real world.

## Methods

### Plant material

The plant genotype Ey15-2 was originally collected from Eyach (Germany), near the sites in which *Pseudomonas* was isolated [16]. It represents a genetic background common to this region [41]. Seeds were sterilized by overnight incubation at −80 °C, followed by shaking of seeds for 5–15 min in a solution containing 75% EtOH and 0.5% Triton-X-100, washing with 95% EtOH and drying in a laminar flow hood). Seeds were stratified in the dark at 4 °C for seven days, on ½ MS-agar media including vitamins and MES buffer (M0255; Duchefa, Germany). Plants were grown in 1.8 ml ½ MS-agar in 24-well plates. All plants were grown on long days (16 h of light) at 23 °C, in plant-growth chambers (CLF CU-36L5, CLF Plant Climatics, Germany).

### Plant protection assay

*Pseudomonas* strains were from a local collection [16], except for *Pseudomonas syringae* pv. *tomato* DC3000, which was taken from lab stocks. All bacterial treatments were diluted to a concentration of OD_600_ = 0.01 per strain. Thus, in co-infections with two strains the total bacterial concentration was OD_600_ = 0.02,), while in infections with individual strains the total concentration was OD_600_ = 0.01. Bacteria-free control was 10 mM MgSO_4_.

Bacterial inocula were prepared as follows: The relevant isolates were grown overnight in Lysogeny broth (LB) and 10 mg/ml nitrofurantoin, an antibiotic to which our *Pseudomonas* isolates are resistant. Cultures were diluted 1:10 in the following morning and grown for 3 additional hours until they entered the log phase. Subsequently, bacteria were pelleted at 3500 g, resuspended in 10 mM MgSO_4_, pelleted again at 3500 g to remove residual LB, and resuspended again in 10 mM MgSO_4_ to a concentration of OD_600_ = 0.02, creating a stock solution for each isolate for subsequent mixtures with either 10 mM MgSO_4_ or a solution containing another strain, in both cases resulting in OD_600_ = 0.01 per strain.

All plants were treated with the relevant inocula 10 days after stratification. Infections were done by drip-inoculation, pipetting 100 μl onto the whole rosette, and plates were subsequently sealed using Micropore tape (3 M, Germany). The plants were photographed on the day of infection (before the actual infection), one day post-infection (day 1), and consecutively from day 4 to day 7 post-infection, using a tripod-mounted Canon PowerShot G12 digital camera and built-in flash. Lids were kept closed to maintain sterility, while a backlight was used to avoid reflections due to the flashlight. Green pixel counts were approximated as described [42]. In brief, individual plants were segmented from the background using lab color space thresholds, followed by morphology-based noise removal. Lastly, GrabCut postprocessing was applied, resulting in a list of plant IDs and the corresponding green pixel count. The script was written in Python 3.6, bash using OpenVN 3.1.0 and scikit-image 0.13.0.

### Gene knockout in *Pseudomonas*

Genes were deleted in strain p5.F2 [16]. First, 300 bp flanking the relevant genes were extracted from the published p5.F2 genome [16]. Restriction sites (XhoI/AvrII/PmeI/PmlI) were added *in silico* between the two fragments of 300 bp flanking sequences, and the resulting sequence was synthesized and cloned into the pDEST2T18ms vector (https://www.addgene.org/72647/) by Twist Bioscience (USA). The full sequence list can be found in Supplementary Table S3.

The Gm^R^ marker for gentamicin resistance was amplified from the template pUC18-mini-Tn7T-Gm-lux (https://www.addgene.org/64963/) with primers 5’ -ccgagctcatgcatgatcg-3’ and 5’-ccggacgatcgaattgggg-3’ in a 25 μL reaction containing 0.25 μL Q5 high-fidelity DNA polymerase (New England Biolabs, USA), 1x Q5 5x reaction buffer, 0.08 μM forward and 0.16 μM of reverse (tagging) primer and 200 μM dNTP. PCR was carried out for 98 °C for 30 s, followed by 30 cycles of 98 °C for 15 s, 58 °C for 20 s, 72 °C for 2 min, and a final extension step of 72 °C for 2 min. The amplified fragment was gel-purified (GeneJET Gel Extraction Kit; Thermo Scientific, USA), and ligated into the PmeI site between the synthesized 300 bp flanking regions using T4 DNA-Ligase (Thermo Fisher Scientific, USA), following the standard restriction-ligation protocol as instructed by the manufacturer. The resulting fragment for each gene was 300 bp 5’ sequence–Gm^R^–300 bp 3’ sequence, cloned into a pDEST2T18ms vector. DH5α competent *E. coli* cells were transformed with the ligation product and subsequently plated on selective Lysogeny broth (LB) agar (1.75%) with gentamicin (10 µg/ml) and tetracycline (5 ng/ml). Bacterial colonies were picked and used as a template for colony PCR using primers matching the foreign DNA (primers in Supplementary Table S4) to identify successful transformants. PCR reactions of 25 μL contained 0.25 μL Q5 high-fidelity DNA polymerase (New England Biolabs, USA), 1x Q5 5x reaction buffer, 0.08 μM forward and 0.16 μM of reverse (tagging) primer and 200 μM dNTP. PCR was carried out for 98 °C for 30 s, followed by 30 cycles of 98 °C for 30 s, appropriate annealing temperature for the primers used (Supplementary Table S4) for 30 s, 72 °C for 2 min, and a final extension at 72 °C for 2 min. The same conditions were used for all following colony PCRs.

Recipient strain p5.F2 was subjected to triparental mating with positive DH5α donors, carrying the pDEST2T18ms with the relevant insert for each gene, and HB101 E. coli, carrying the plasmid pRK2013 that facilitates mobilization, as helper strain. Cultures of the donor, recipient and helper strains were grown overnight with shaking, at 37 °C for donor and helper, and 28 °C for the recipient, in LB with the relevant antibiotics (10 µg/ml gentamicin and 5 ng/ml tetracycline for the donor, 100 µg/ml nitrofurantoin for the recipient and 50 µg/ml kanamycin for the helper). The following morning, cultures were diluted 1:10 and grown for 2–4 additional hours. The three strains were then mixed in a single tube, followed by immediate centrifugation (3000 g), removal of supernatant and resuspension in 1 ml LB. The washing procedure was repeated, and cells were resuspended in 100 µl LB. The cell mixture was inoculated onto LB plates to allow for mating and incubated for 72 h at 28 °C. The resulting cell mass was scraped off into 500 µl 10 mM MgSO_4_ and centrifuged for 2 min at 3500 g. The supernatant was removed, and the cells were resuspended in 1 ml of 10 mM MgSO_4_. A 100 µl aliquot of the resulting mixture was plated onto LB-agar selective media including 100 µl/ml nitrofurantoin, gentamicin 10 µg/ml and tetracycline 5 ng/ml. The plates were incubated at 28 °C for 2–3 days until visible colonies appeared. Colony-PCR (primers in Supplementary Table S4) was used to identify successful p5.F2 transformants, and to validate that the native gene was replaced by the Gm^R^ cassette by homologous recombination. Successful knockout was deduced from the size of the PCR product (Supplementary Table S4), the amplified fragment was gel-purified (GeneJET Gel Extraction Kit; Thermo Scientific, USA) and Sanger-sequenced to confirm the presence of the expected Gm^R^ marker. Positive transformants were plated on media with 5% sucrose and 10 µg/ml gentamicin, to eliminate the pDEST2T18ms plasmid, which carries the sucrose counterselection marker SacB. Loss of SacB was confirmed by colony-PCR (same reaction and conditions as for Gm^R^ PCR, primers in Supplementary Table S4) and gel electrophoresis, and re-examined for the absence of the native gene and for the presence of Gm^R^.

As the knockout mutants are Gm^R^, we also generated a Gm^R^ wild-type derivative, ensuring a similar metabolic burden as in the mutants. *Pseudomonas* strain p5.F2 was transformed with the plasmid pUC18T-mini-Tn7T-Gm-lux (https://www.addgene.org/64963/), using the mini-Tn7 transformation protocol [43]. In brief, bacteria went through several sucrose washes, and subsequently were transformed with the plasmid by electroporation, together with the helper plasmid pTNS2 (https://www.addgene.org/64968/). Insertion is a single copy into a locus considered as neutral [43].

### Iron sensitivity assay

Growth sensitivity to iron levels was assayed as described [44]. Briefly, each strain was grown in 200 µl LB in the presence of increasing 2,2’-dipyridyl concentration, ranging from 0 to 300 nM. Bacteria were diluted to a starting concentration of OD_600_ = 0.05 in 96-well plates (Greiner Bio One, Austria). Plates were incubated in a plate reader (Robot Tecan Infinite M200; Tecan Life Sciences, Switzerland) at 28 °C while shaking for 10 h, and OD_600_ was measured in 30 min intervals.

### Phylogenetic analysis

All maximum likelihood phylogenies were based on the core genome (ortholog presence > 70%) of the relevant strains in the analysis, constructed with IQtree (v1.6.10; using the parameters: -mset LG -st AA -nt 16 -ntmax 16) [45]. Trees were visualized with iTOL [46], except for the combined tree-heatmap plots, in which the R function *ggtree* from the package *ggtree* was used [47].

### Regression analysis

Posterior distributions of the relevant predictors were approximated using the function *stan_glm* in the R package rstanarm [48]. Default settings including prior distribution were used, unless more iterations were required (Rhat > 1.1). The number of iterations was increased until a sufficient number was reached (Rhat < 1.1). In all figures, the median and 95% credible intervals from 2.5% to 97.5% of the posterior distribution are presented. The response variable and predictors, as well as the reference for comparison, are explained in the figure legend for each analysis.

### Bacterial genome-wide association with plant protection

Gene orthology clusters as well as the phyletic pattern of gene presence/absence (P/A) for each strain used in this study, were from [16]. Associations between gene orthologs and plant growth used treeWAS [19], a tool for bacterial GWAS. Phyletic pattern and phenotypic measurements for all strains can be found in Supplementary Data 1. The core-genome-based phylogeny used for treeWAS can be found in Supplementary Data 2.

Four different sets of strains were used, in four separate analyses: All strains; ATUE2; ATUE3; ATUE4. In each set, the resulting maximum likelihood phylogeny was used in treeWAS. To gain higher sensitivity, each set of strains was analyzed using four different metrics of plant growth. All significant hits per set of strains were combined, and the number of tests in which an ortholog resulted as significant was counted (Supplementary Table S2). The four plant growth metrics that we used were median change in green pixels between the end of the experiment 7 days post-infection and the day of infection (median growth); a Bayesian-derived approximation of median growth (cdl50, the posterior distribution median of median growth); a binary categorization based on cdl50 (cdl = < 0, i.e., zero growth or decline, and cdl > 0, i.e., any amount of growth); and the area under the curve while accounting for all sampled time points (0–1, 4–7 days post-infection), as the value of *auc_l* after running the R function *growthcurver* [49]. The four metrics are detailed in the legend of Supplementary Table S2.

Heatmaps were produced and combined with phylogenetic trees using the R function *gheatmap* from the package *ggtree* [47].

To test for physical proximity between significant genes, that is, whether they potentially formed genomic islands, we examined the location of the corresponding genes in strains that had all of these genes. We accounted for gene ID provided by Prokka [50], which includes the order of genes within a contig. For example, gene_1 and gene_3 are separated on contig_X only by gene_2. Only genes that were found on the same contig and that were separated by no more than one gene were considered as consecutive. All genes behaved in a congruent manner across strains examined. We further validated the proximity of genes by counting the number of bases differing from each consecutive pair of genes, confirming that they were at most hundreds of bp apart.

### Gene ortholog annotation

To assign the potential function to each of the proteins encoded by the studied genes we first used the multiple sequence alignment of the protein orthology group to build a representative HMM model using HMMER (version 3.2.1). Next, the resulting HMMs were used to search for homologues proteins using *hmmsearch* against the UniProt Knowledgebase (Swiss-Prot+TrEMBL); release 2020_05. The function of the best significant hit, which covered at least 80% of the query protein, was transferred to the query protein. Annotations of each orthology group can be found in Supplementary Data 3.

### Analysis of growth in iron-sensitivity assay

The growth of all bacteria was analyzed using the R function *SummarizeGrowthByPlate* from the Growthcurver R package [49]. The logarithmic area under the curve, *auc_l*, was extracted and compared between the wild-type, mutant strains of interest and the focal pathogen.

### Statistical analysis

Statistical analyses were performed using R (version 3.5.1), unless mentioned otherwise.

## Supplementary information

Supplementary Figures S1–S10

Supplementary Table 1

Supplementary Table 2

Supplementary Table 3

Supplementary Table 4

Dataset 1

Dataset 3

Dataset 2

## Data Availability

Knockout mutants are freely available from the authors.

## Code availability

The code for all analyses including the GWAS, is available at https://github.com/orshalevsk/GWAS_project.
