# Supplementary material for: Commensal Pseudomonas protect Arabidopsis thaliana from a coexisting pathogen via multiple lineage-dependent mechanisms
Source: ISME J. 2021 Dec 11;16(5):1235–44. doi: 10.1038/s41396-021-01168-6 (PMC9038753; doi:10.1038/s41396-021-01168-6)

Supplementary Material for:

Commensal *Pseudomonas* protect *Arabidopsis thaliana* from a coexisting pathogen via multiple lineage-dependent mechanisms

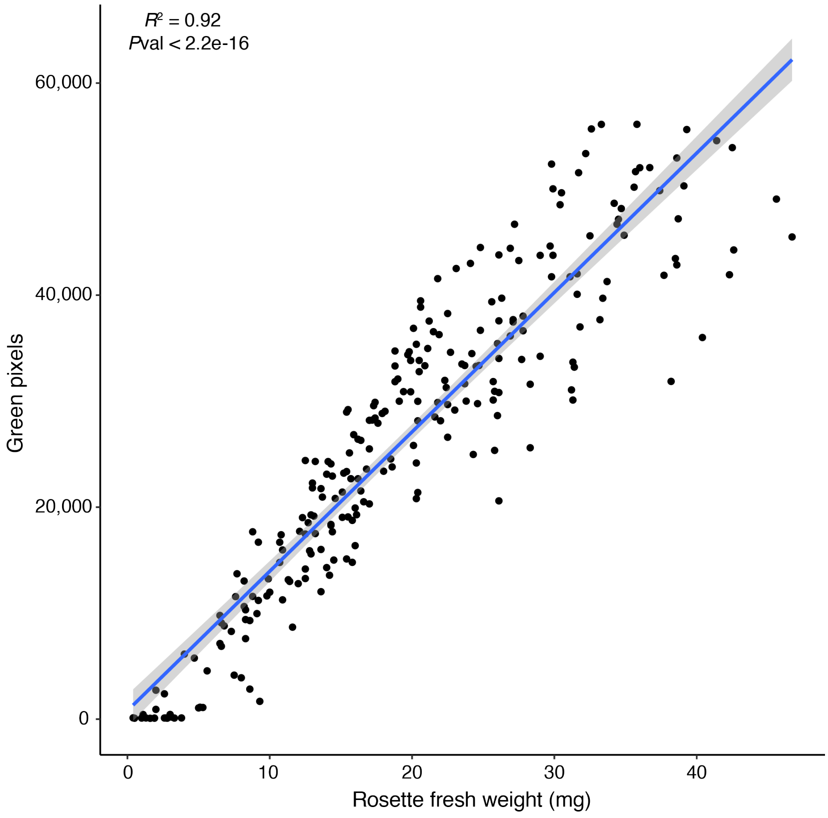

Figure S1. Correlation between green pixels count and rosette fresh weight. Plants were randomly sampled 7 days post-infection (end of experiment) for weight measurement. Green pixels count retrieved from the same day of sampling. Blue indicates the regression line, and the shaded area indicates 95% confidence interval. n=251.

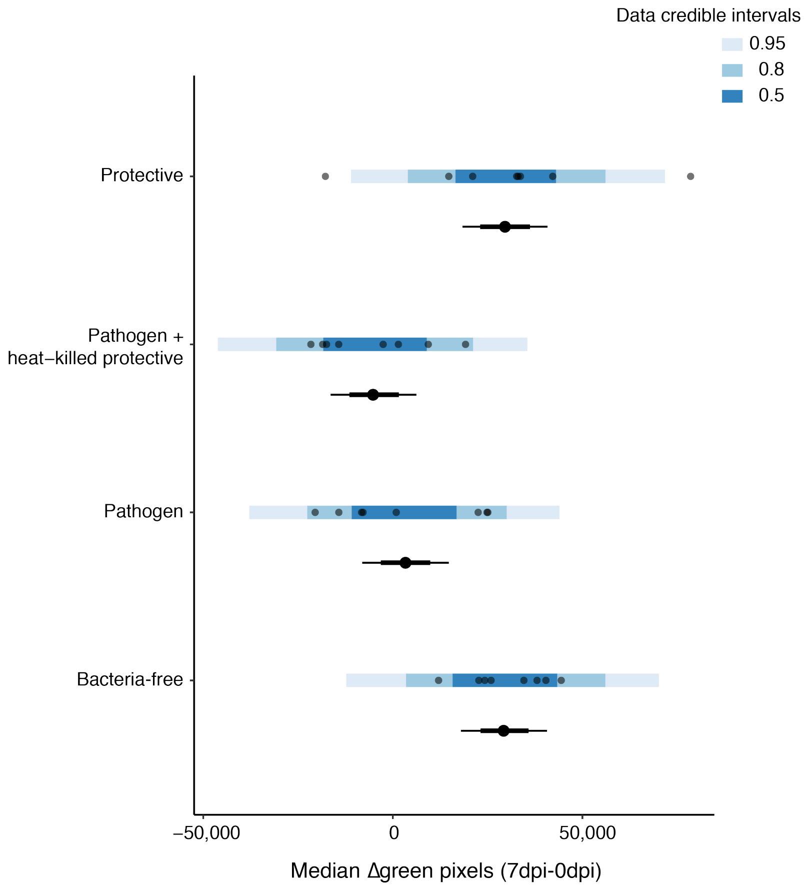

Figure S2. Plant growth in control treatments. Plant growth in control treatments: Bacteria-free, pathogen and co-infections of the pathogen with the protective strain and heat-killed protective strain. Growth was measured by the median change in green pixels between day 7 post-infection to the day of infection. In each treatment, raw data is described by the dots at the top panel, and the shades of blue indicate posterior predictive intervals. The bottom panel of each treatment manifests the mean growth: Dot indicates the median of the credible interval, while the vertical line indicates 95% credible interval. n=8.

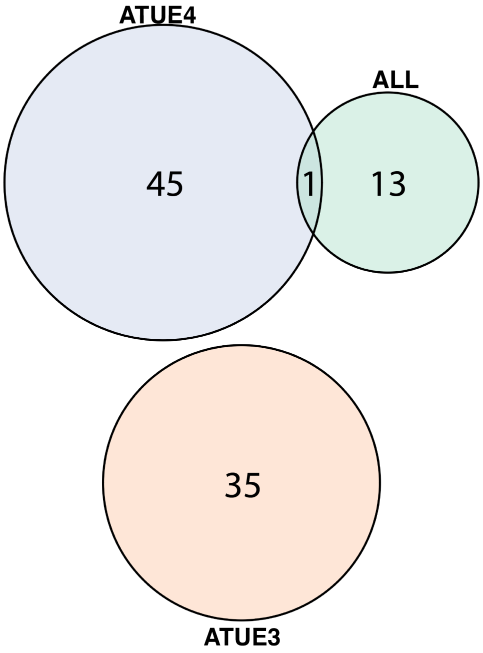

Figure S3. Euler diagram of significant genes in three strain sets. Significant genes, as determined by treeWAS analysis [19] after including all strains, and the subsets of ATUE2, ATUE3 and ATUE4. Four analyses were conducted, using four different metrics to manifest plant growth (**Table S2**; Methods). Only significant hits with positive Spearman rank correlation coefficient were considered. No significant hit with positive correlation was found after analyzing only the ATUE2 subset.

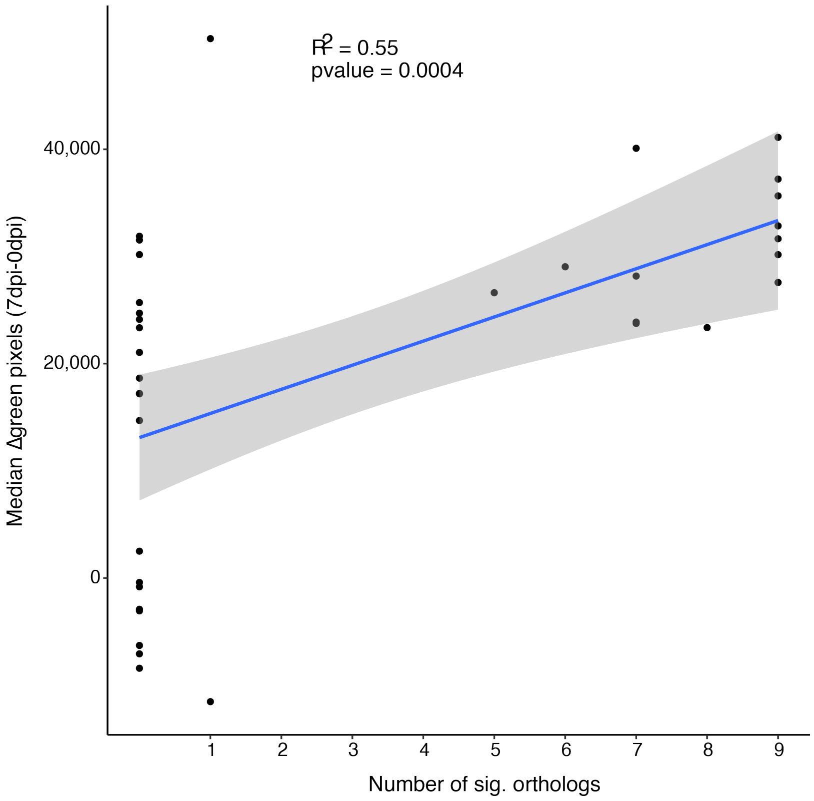

Figure S4. Correlation between median plant growth and the number of significant genes presented in a given strain, in the ATUE2 subset. Plant growth was measured by the median change in green pixels between day 7 post infection to the day of infection. Only the curated gene set was considered, as presented in **Figure 3A**. Blue indicates the regression line, and the shaded area indicates 95% confidence interval. n=8 per each of 36 strains.

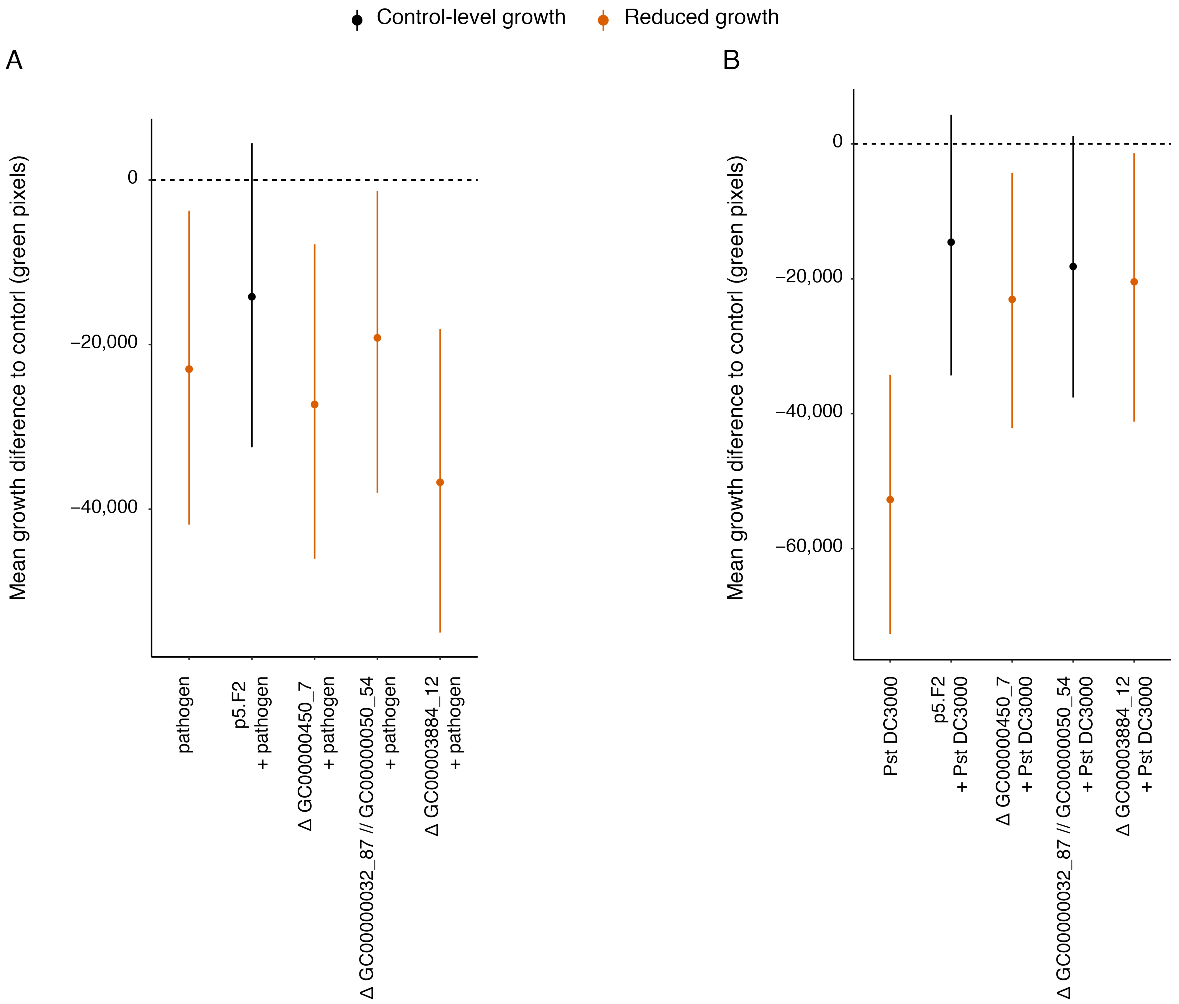

Figure S5. Co-infections of the wild type p5.F2, and each of three tested knockout mutants with (A) ATUE5 pathogen and (B) *Pseudomonas syringae* pv. *tomato* DC3000 (*Pst*). Mean plant growth difference to control (bacteria-free treatment) was calculated using the model [Δgrowth ~ treatment]. Control signified by the dashed horizontal line. Growth was measured as the change in green pixels between the day of infection until day 7 days later. Vertical lines indicate 95% credible intervals of the mean, while dots indicate the median. Colors indicate the growth difference of co-infections to control, as determined by the overlap between the baseline and 95% credible intervals. n=20. Related to **Figure 4**.

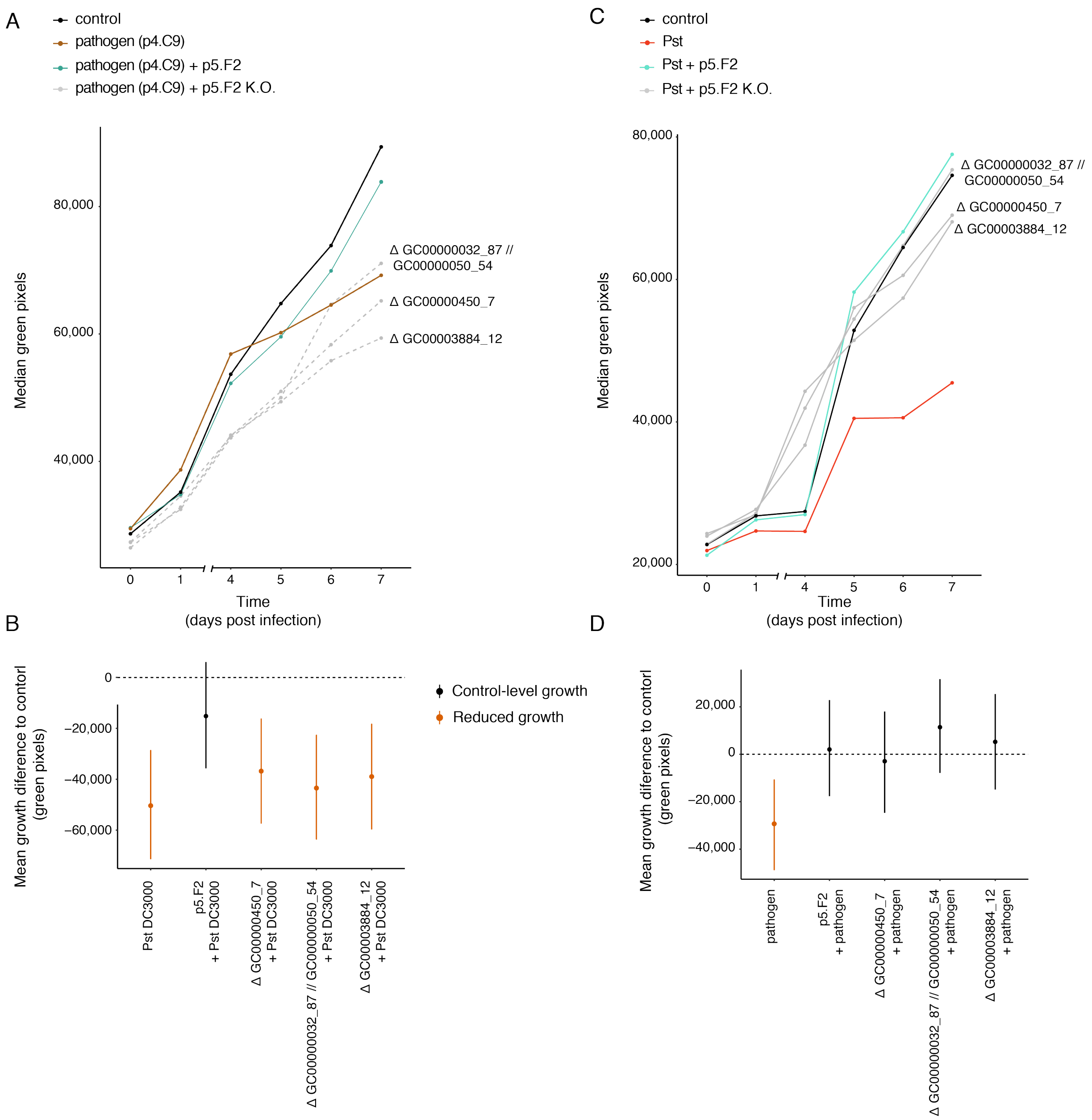

Figure S6. Co-infections of the wild type p5.F2, and each of three non-protective knockout mutants with (A-B) ATUE5 pathogen and (C-D) *Pseudomonas syringae* pv. *tomato* DC3000 (*Pst*). Additional experiment. A and **C**, Daily median of plant green pixels. Dashed lines note treatments comprising p5.F2 knockout mutants that lost their ability to protect the plant, as inferred from the statistical analysis in panels B and D. Plants were assessed daily by imaging. **B** and **D**, Mean plant growth difference to control (bacteria-free treatment) after a relevant *Pseudomonas* infection, as calculated using the model [Δgrowth ~ treatment]. Control signified by the dashed horizontal line. Vertical lines indicate 95% credible intervals of the mean, while dots indicate the median. Colors indicate the growth difference of co-infections to control, as determined by the overlap between the baseline and 95% credible intervals. n=17-20.

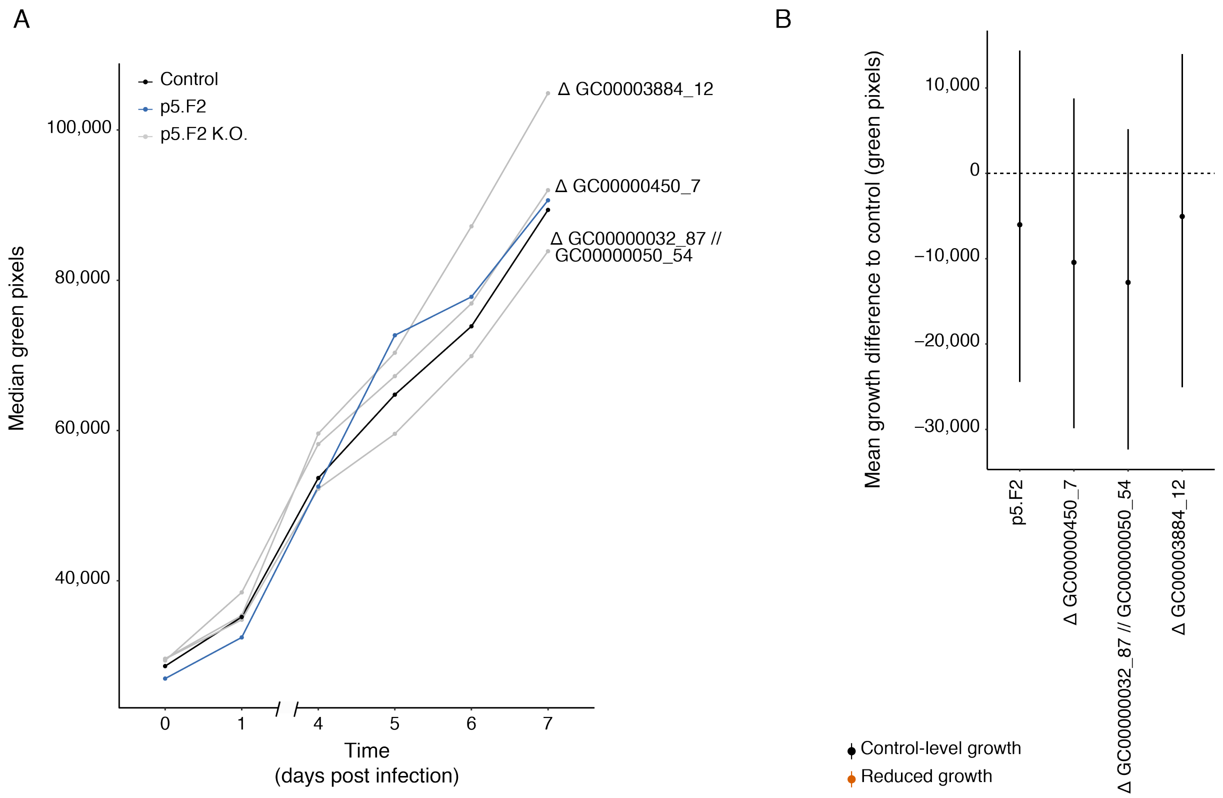

Figure S7. Individual infections with the wild type and three non-protective knockout mutants. A. Daily median of plant green pixels after treatment with control, p5.F2 and the three non-protective knockout mutants ('p5.F2 K.O.'). Plants were assessed daily by imaging. n=20. **B.** Mean plant growth difference to control after treating plants with the protective strain p5.F2 and the three p5.F2 knockout that lost their protective ability ('Δ GC00000032_87 // GC00000050_54', 'Δ GC00000450_7' and 'Δ GC00003884_12'). Vertical lines indicate 95% credible intervals of the mean, while dots indicate the median. The dashed horizontal line signifies the baseline, which manifests plant growth after bacteria free treatment (i.e. 'control'). Colors indicate the growth difference of co-infections to control, as determined by the overlap between the baseline and 95% credible intervals. n=20.

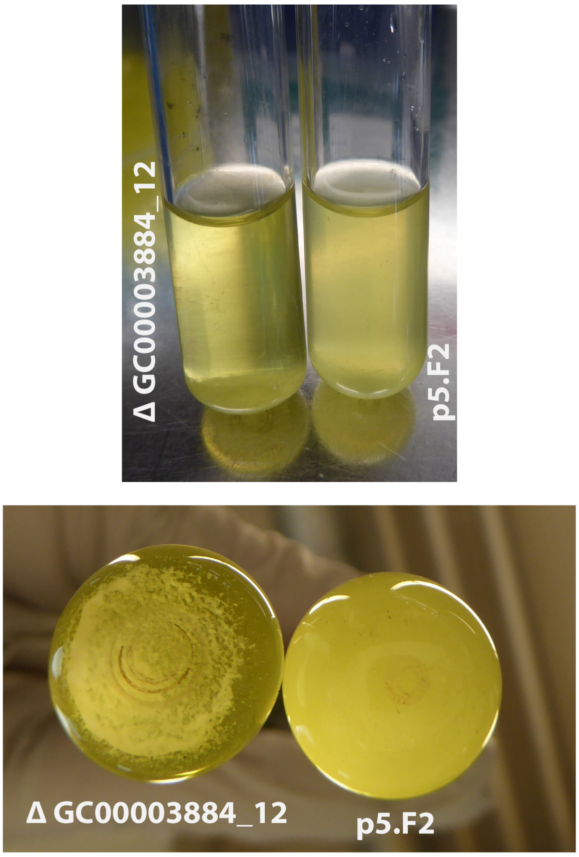

Figure S8. Representative images of LB-grown p5.F2 and its knockout mutant ΔGC00003884_12, one hour after static incubation. Experiments performed three times with similar results.

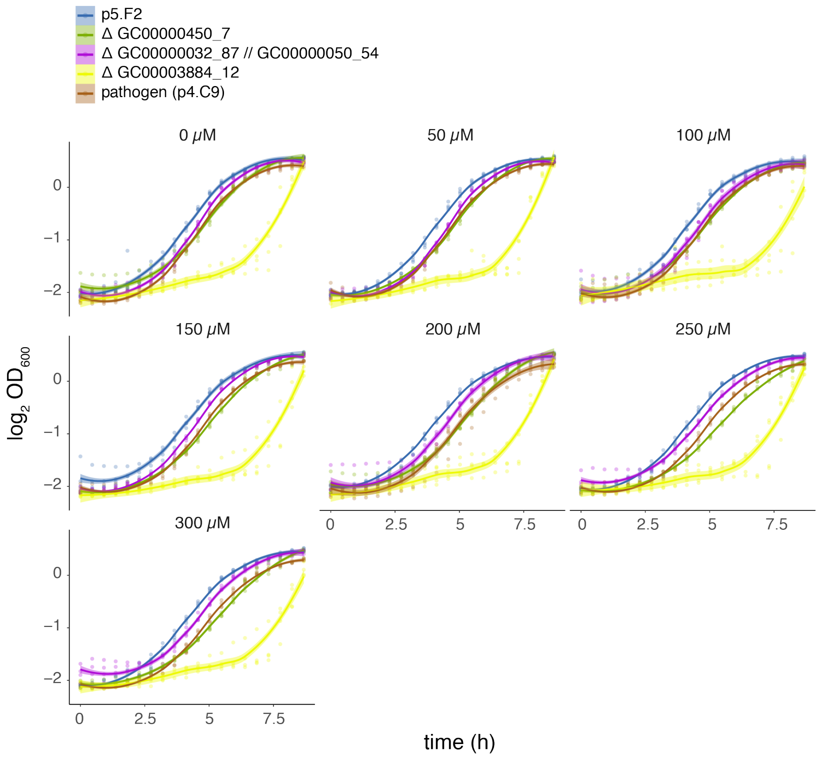

Figure S9. Growth curves of the wild type strain p5.F2, focal three knockout mutants and the pathogen in increasing 2,2'-bipyridine concentration. Bacteria were grown in LB medium, supplemented with seven different 2,2'-bipyridine concentrations (0, 50, 100, 150, 200, 250 and 300 nM). Growth was monitored for 10 hours in 30 minutes intervals. Shaded area indicates 95% confidence intervals of the regression curve. n=4.

Figure S10. Regression analysis of bacterial growth as a function of 2,2'- bipyridine concentration. The growth of the two p5.F2 knockout mutants ('Δ GC00000032_87 // GC00000050_54' and 'Δ GC00000450_7') and the pathogen were compared to the wild type protective strain p5.F2. The logistic area under the growth curve was extracted as a proxy for bacterial growth. The linear model {growth ~ strain*chelator} was analyzed, while 'chelator' states 2,2'- bipyridine concentration. A. Growth difference without supplementation of 2,2'-bipyridine (thus, the difference to p5.F2 of the variable 'strain' while 'chelator' is zero, i.e. the intersection with the y axis). B. Difference in the effect of 2,2'-bipyridine on growth, by strain (thus, the difference to p5.F2 of the interaction between the variables 'strain' and 'chelator', i.e. the slope of each strain). Vertical lines indicate 95% credible intervals of the mean, while dots indicate the median. Dashed horizontal lines signify the baseline, which manifests the wild type strain p5.F2. Colors indicate the difference to p5.F2, as determined by the overlap between the baseline and 95% credible intervals. n=4. Related to figure 4B.

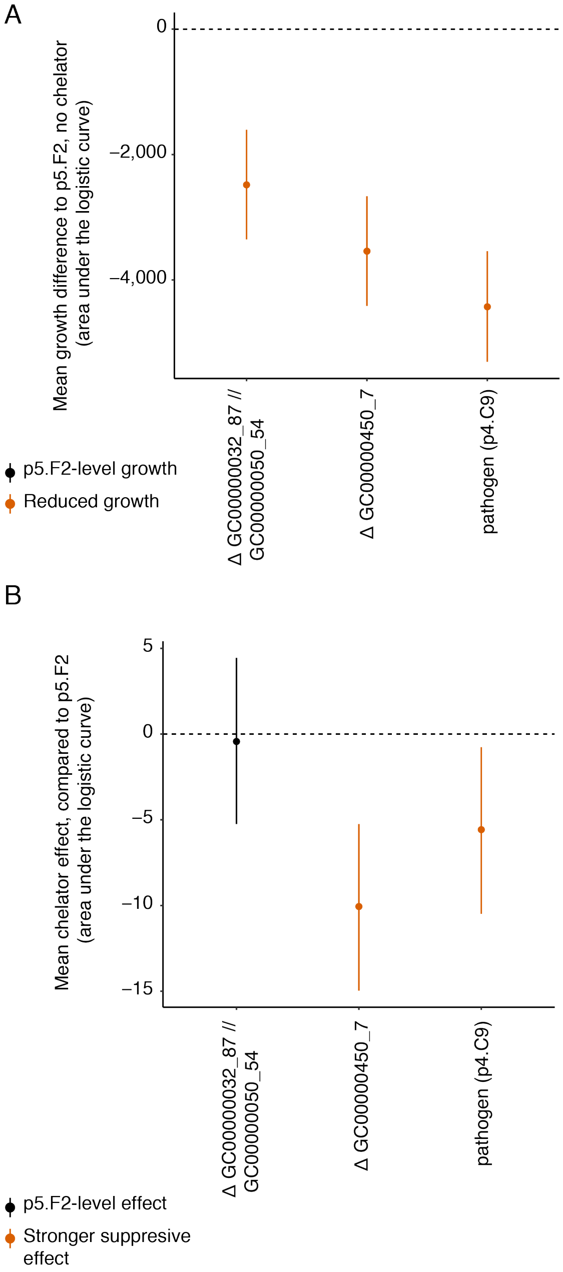

Supplement: Supplementary file 1 — Supplementary Figures S1–S10 [file 41396_2021_1168_MOESM1_ESM.pdf]
